# Genomic Signatures in Microbes—Properties and Applications

**DOI:** 10.1100/tsw.2011.70

**Published:** 2011-03-22

**Authors:** Jon Bohlin

**Affiliations:** Department of Food Safety and Infection Biology, Norwegian School of Veterinary Science, Oslo, Norway

**Keywords:** genomics signatures, microbes, pathogenicity islands, nonhomologous DNA sequence comparisons

## Abstract

The ratio of genomic oligonucleotide frequencies relative to the mean genomic AT/GC content has been shown to be similar for closely related species and, therefore, said to reflect a “genomic signature”. The genomic signature has been found to be more similar within genomes than between closely related genomes. Furthermore, genomic signatures of closely related organisms are, in turn, more similar than more distantly related organisms. Since the genomic signature is remarkably stable within a genome, it can be extracted from only a fraction of the genomic DNA sequence. Genomic signatures, therefore, have many applications. The most notable examples include recognition of pathogenicity islands in microbial genomes and identification of hosts from arbitrary DNA sequences, the latter being of great importance in metagenomics. What shapes the genomic signature in microbial DNA has been readily discussed, but difficult to pinpoint exactly. Most attempts so far have mainly focused on correlations from *in silico* data. This mini-review seeks to summarize possible influences shaping the genomic signature and to survey a set of applications.

## BACKGROUND

Biochemical experiments conducted in the early 1960s revealed remarkable species-specific patterns as measured using dinucleotide frequencies divided by corresponding mononucleotide frequencies, i.e., $f(XY)/f(X)f(Y)$, X,Y={A,G,C,T(U)}, in genomic DNA[1]. Subsequent investigations well into the late 1970s strengthened the notion of species-specific patterns in genomic DNA[2]. However, the number of genomic DNA molecules available for analyses were from a limited number of incompletely sequenced species, making any general inference speculative. Although many extraordinary patterns in genomic DNA were further discovered during the 1980s, little progress was made in terms of the species-specific signal discovered by Josse et al.[1]. An increasing number of available genomic DNA sequences in the early 1990s allowed for a more thorough comparison of DNA sequences between species. Since the species-specific genomic patterns discovered in the early 1960s were still found to hold for all sequences examined, Karlin and Burge concluded that a "genomic signature" must be present in all living species[3]. The advent of the first bacterial full genome sequence available, *Haemophilus influenzae*, also made it possible to examine a whole microbial genome[4]. With additional microbial genomes fully sequenced, it was found that the signature was more or less the same throughout the genome when the signal of relatively small pieces of genomic DNA, i.e., 10–50 kbp, were compared to the whole genomic DNA sequence-based signal[5]. In other words, only minor signature differences were detected between smaller pieces of DNA and the corresponding whole genome DNA sequence. The signature differences were found to vary less within than between species, at least for bacterial genomes. A more elaborate investigation of genomic signature variations in the *Helicobacter pylori* genome revealed regions of unusually large signature differences[6]. Variations in genomic GC content within a bacterial genome are usually also reflected by the genomic signature[6]. In the *H. pylori* genome, however, several regions were detected with few or no differences in base composition, but there were large variations in the genomic signature[6]. Further examination of these regions revealed that they contained genes associated with virulence factors. Although considerable research has been carried out on the genomic signature, the cause of this signal has been difficult to assess properly because of its genome-wide character and the inherent difficulty of perturbation[5,7,8,9,10]. The purpose of this mini-review is to review what is known so far about genomic signatures and to examine a set of applications in prokaryotes.

## METHODS TO EXTRACT THE GENOMIC SIGNATURE

The genomic signature was originally computed by calculating all dinucleotide frequencies (16 in total) of a genomic DNA sequence divided by the corresponding nucleotide frequencies constituting the dinucleotide[1,3]. In other words, if $f_A(.)$ designates the frequency of a nucleotide X or dinucleotide XY, where X and Y can be any combination of nucleotides (A[denine], G[uanine], C[ytosine], T[hymine] or U[racil] for RNA), from DNA sequence A, the genomic signature is found by calculating the ratios $\rho = f_A(XY)/f_A(X)f_A(Y)$ for every possible combination of dinucleotides XY. To compute the signature difference between two DNA sequences, A and B, a difference measure is typically used. Karlin et al.[3] described the signature difference between two DNA sequences by using the average absolute magnitude measure:

$$\delta^*(A,B) = \frac{1}{n}\sum_{XY}\left|f_A(XY) - f_B(XY)\right|$$

where $n$ is the number of possible dinucleotide combinations ($n$ = 16 for dinucleotides, $n$ = 256 for tetranucleotides, etc.). It is sufficient to carry out analyses on one strand[7]. Although this difference measure is most often used[8,9,11], other measures do also exist. Examples include the empirical variance formula[12], $R^2$ from linear regression[7], the Pearson correlation formula[13], chi square goodness of fit[14], and more. Wu and coworkers provide an overview of different measures that can be used to compare signatures[15]. The choice of measure depends on the application. For instance, when searching for pathogenicity islands within a bacterial genome, the Pearson correlation measure might be more intuitive in the sense that it gives a more familiar quantity, ranging from 0 (no similarity) to 1 (complete similarity). The same is true for the regression method using the “coefficient of determination” $R^2$ instead of the Pearson correlation measure. Both linear regression and Pearson correlation methods are linear. This means that both these measures do not give meaningful results for more dissimilar DNA sequences. Typically, quantities below $r = 0.8$ ($R^2 = 0.6$) give little information[13]. The Mahalanobis measure is very general and allows for a correlation structure accounting for dependencies between the oligonucleotides to be specified[16]. This measure is therefore more complicated to compute if a special correlation matrix is assumed. It is, however, the most general measure[16] and is equivalent to the Pearson correlation measure if a unity matrix assuming independence between the oligonucleotides is specified as the correlation structure. Nevertheless, the drawback of all such measures is that they compress all computed ratios into one quantity. In effect, this is an irreversible compression with loss of information. There is only one example known to the author where the measure has been altogether dropped and cluster analysis been carried out directly on the computed ratios for all DNA sequences to assess phylogeny[17]. Without a distance measure, there will be as many columns as there are computed ratios. This means 16 columns for dinucleotide frequencies, 64 for trinucleotide frequencies, 256 columns for tetranucleotide frequencies, etc. These ratios can be plotted using a heatmap to reveal species-specific patterns. Such analyses were first carried out to investigate occurrences of pathogenicity islands and foreign DNA within microbial genomes[12,18,19], but have subsequently been used to compare genomic DNA sequences between species[17]. Comparing genomic DNA sequences directly with cluster analysis offers an additional advantage over measure-based methods by providing a more detailed visual representation of each genome, especially with the use of heatmaps. Although the genomic signatures in this review are predominantly computed by the $0^{th}$-order Markov chain model[20], by dividing the oligonucleotide frequencies with the corresponding nucleotide frequencies, i.e., $\rho = f_A(XYYZ)/f_A(X)f_A(Y)f_A(Y)f_A(Z)$ in the notation used above, other more advanced methods have been applied. Most notable of these is the second-order Markov chain model[5,7,21,22] and, rarely, a first-order Markov chain model[14,23]. Extensive study of these more advanced Markov chain models has not revealed any advantage over the simple $0^{th}$-order Markov chain model[7,13,23]. It should be mentioned, however, that increasing the order of the Markov chain improves the estimation of larger oligonucleotide frequencies, implying that prokaryotic genomes are short-range correlated to a large degree[23]. In other words, genomic base composition in prokaryotes is influenced by neighboring nucleotides to a large extent.

## WHAT CAUSES THE GENOMIC SIGNATURE?

Although the genomic signature was first revealed more than 40 years ago, its cause is difficult to understand, most likely due to many contributing factors[8,24]. The availability of continuously more genomic sequences has made it possible, however, to examine the matter in greater depth using *in silico*–based methods. Karlin et al. suggested that certain DNA replication- or repair-based enzymes might be associated with the genomic signal in genomic DNA[5], and some support for this has been found. Zhao and coworkers found associations between the presence of a Pol III $\alpha$ subunit and GC content variability in microbial genomes[25]. The similar signatures may therefore be explained, at least in part, by the presumption that phylogenetically closely related organisms are more likely to have the same or similar proofreading enzymes. More support for this is found in the amelioration of bacterial genomes, where the genomic replication and repair machinery modifies the base composition of foreign DNA, for example, at the variable third codon position, to progressively resemble that of the host genome[26]. Karlin and coworkers also predicted that DNA structural features would influence the genomic signature[5]. Indeed, results are readily available that point to a possible association between DNA structural features and genomic base composition[20], which, in turn, will affect the genomic signature of the organism. While it was originally thought that genomic GC content would not affect the genomic signature because of the way it was calculated[3], an association with genomic GC content was later found[24]. For instance, GC-rich genomes were found to be more homogeneous than AT-rich genomes[23]. Similarly, no signature differences were first detected between protein coding and noncoding regions[5]. However, by using tetranucleotide frequencies instead of dinucleotide frequencies, significant signature differences were found between coding and noncoding regions[7]. Thus, there were clear differences between the signatures in AT- and GC-rich genomes, and coding and noncoding regions. The reason for this is not known, but strains with AT-rich genomes are often associated with organisms living an intracellular life with genome decay leading to smaller genomes with fewer genes[27,28]. Species with GC-rich genomes are usually found in the soil and tend to have complex life styles, with large genomes containing many genes[29,30,31]. More energy is, in general, required to destack GC-rich dinucleotides than AT-rich dinucleotides; therefore, the genomes of GC-rich organisms are both more stable, but also more expensive to maintain in terms of energy cost compared to AT-rich genomes[32]. One of the contributing factors between signature differences in AT- and GC-rich genomes is that AT-rich genomes appear to have been subjected to mutational bias possibly due to loss of repair genes[33] or relaxed selective pressures[34]. This may be related to mutational bias being associated with cytosine to thymine deamination[35]. Environment thus appears to have an effect, although possibly indirect, on the genomic signature[24]. GC-rich genomes are more nitrogen rich, and this nitrogen can be taken from the soil and vegetation[36]. On the other hand, an association between extreme environments, i.e., environments with unusual high or low temperatures, salt concentrations, pressure, etc., and base composition has been more difficult to establish. This might be due to the linear methods used to examine possibly highly nonlinear phenomena[37]. For instance, whether genomic GC content can be associated with growth temperature or aerobiosis is still disputed[23,38,39,40,41,42,43,44]. Regression analysis revealed that environment and other factors were associated with the genomic signature, but phylogeny and, most of all, GC content were by far the strongest associations[24].

## APPLICATIONS

A species-specific signal in microbial DNA sequences offers many applications. Originally, it was suggested that genomic signatures could be used for phylogenetic classifications and nonhomologous sequence comparisons[3,9,45]. Not only have many other applications subsequently surfaced[13,46,47,48], but genomic signatures have also provided deeper insight into biological and evolutionary processes. Nevertheless, the possibility of comparing nonhomologous genes, read sequence quality, global motif identification, and identifying organisms from relatively small arbitrary DNA sequences are features that hold promise in exploring the numerous DNA sequences that are forthcoming at an exploding rate[14,46,48,49,50,51]. The ability to perform nonhomologous comparisons and host identification from arbitrary DNA sequences can be used to search genomic DNA sequences for exogenous or anomalous DNA in microbial genomes, such as horizontally transferred DNA, pathogenicity islands, and bacteriophages in microbes[6,11,12,18,20,23,52,53,54]. Considerable effort has been spent examining the ability of genomic signatures to detect exogenously acquired DNA. While Karlin considered genomic signatures to be superior to standard GC content[6], Baran and Ko found that GC content appeared to be a more reliable method with fewer false positives[11]. It is nevertheless clear that the genomic signature method can detect foreign DNA not easily distinguishable by observing intragenomic variations in GC content[6]. Although Baran and Ko question whether genomic signatures give an unjustifiable number of false-positive foreign DNA regions compared to variation in GC content, their claim has recently been contested. This can be found in a comprehensive comparison of oligomer-based methods for detection of horizontal transfers carried out by Becq et al.[55]. Genomic areas subjected to special selective pressures may also show substantial signature variations compared to the host[11].

Using genomic signatures, van Passel and coworkers were able to extract information about the nature of horizontal transfer in a strain of *Vibrio vulnificus*[54]. Not only did their results indicate that the bacterium might have received DNA from multiple hosts, but also, perhaps more importantly, that there have been recursive transfer events from the same donor to the same acceptor. In subsequent work, they also found that plasmids have signatures that differ more than what would be expected from the host DNA sequence[56]. A later study by the author and coworkers showed that there was a significant and positive association between plasmid-host signature similarity and host GC content[13]. In other words, the more GC rich the bacterial genome, the smaller the signature differences between the plasmids and their corresponding hosts. This points to plasmids consisting of foreign DNA, which is especially pronounced in AT-rich genomes. Another possibility is that amelioration rates are faster in GC-rich genomes due to stronger selective pressures[23,34] making the base composition of the plasmids adapt faster to the base compositional patterns of the host genome.

Although signature similarity is low between phages, plasmids, and host genomes[20], the findings of Pride et al. indicate that viruses (both bacteriophages and eukaryotic viruses) coevolve with their hosts[57]. Because of the difficulty in determining the phylogenetic relationships in viral genomes based on phenotypic information, Pride et al. suggested that genomic signatures could be a useful method in viral taxonomy due to the absence of universally conserved marker genes in all viruses such as the 16S rRNA genes found in bacteria[57]. In contrast to Karlin et al. and others, Pride and coworkers used tetranucleotide frequencies instead of dinucleotide frequencies because of allegedly increased precision[7]. They also found that the more advanced maximal-order Markov chain model was inferior to the mathematically simpler $0^{th}$-order Markov method[7]. However, both the $0^{th}$- and maximal-order Markov chain model–based genomic signatures have in common the ability to differentiate between genomic DNA sequences of phylogenetically unrelated hosts with similar GC content[7,22]. Phylogenic classification using genomic DNA sequences is, as mentioned above, one of the main applications of genomic signatures. For a comparison between a set of different genome-based phylogenic methods for microbes, see Coenye et al.[45]. Although some assumptions have been made with respect to the phylogenetic signal found from the genomic signature method, no conclusive systematic analysis with respect to its origins has been undertaken to the best of the author's knowledge. *In silico* examinations carried out by the author and coworkers demonstrated that the genomic signature is first and foremost associated with genomic GC content and phylogeny[24]. Other factors, such as environment, oxygen requirement, and growth temperature, were also found to be significant, although to a considerably lesser extent than genomic GC content and phyla. In this respect, it should be noted that GC content has been found to be more strongly associated with environment than phylogeny[58], implying that the strong association between genomic GC content and the genomic signature might also be influenced by the habitat of the organism. These factors may therefore be the reason that host-integrated foreign DNA sequences have very different signatures than host DNA[20]. Genomic signature variations within genomes are therefore often used as indicators of foreign DNA. This has been examined in several different ways. Fig. 1 illustrates how cluster analysis can be applied to separate host genomic regions from exogenous regions, such as horizontally transferred DNA and pathogenicity islands. Possible sources of DNA uptake may also be separated using cluster analysis (see Fig. 1, right graph) as well as time of integration because of amelioration.

The search for foreign genetic regions is often carried out by comparing the signature from a fraction of the genome, typically 5- to 20-kbp sliding windows, to the whole genome signature[5,11,13,54]. It has been shown that the signature variation within a genome is also associated with several factors, such as phylogeny, aerobiosis, and genomic GC content[23]. In fact, GC-rich genomes have a more stable genomic signature than AT-rich genomes regardless of phyla. Intragenomic signature variation can therefore be considered as a measure of genome homogeneity[23]. The intragenomic stability of the genomic signature was additionally associated with a measure termed oligonucleotide usage variance (OUV)[20,23,59]. This measure estimates the mean genome mutation frequencies by summing the squares of the differences between each possible genomic oligonucleotide frequency and the corresponding frequencies of the individual nucleotides, divided by the sum of total number of oligonucleotide combinations, i.e.,

$$\frac{1}{n}\sum_{XYZW}(f_A(XYZW) - f_A(X)f_A(Y)f_A(Z)f_A(W))^2$$

where $n$ = 256 is the total number of tetranucleotide combinations. High OUV means bias in oligonucleotide usage, and low OUV means similarity between estimated and computed oligonucleotide frequencies. Low OUV is interpreted as increased genomic mutation rates, or lower bias in the oligonucleotide usage since the genomic oligonucleotide frequencies deviate less from the estimated oligonucleotide frequencies[20,23]. The estimated oligonucleotide frequencies are only based on genomic nucleotide frequencies, which imply total independence between each nucleotide in the estimated oligonucleotide. The OUV measure was found to have a direct association with GC content, i.e., GC-rich genomes have higher OUV. AT-rich genomes are therefore, in general, more associated with random genetic

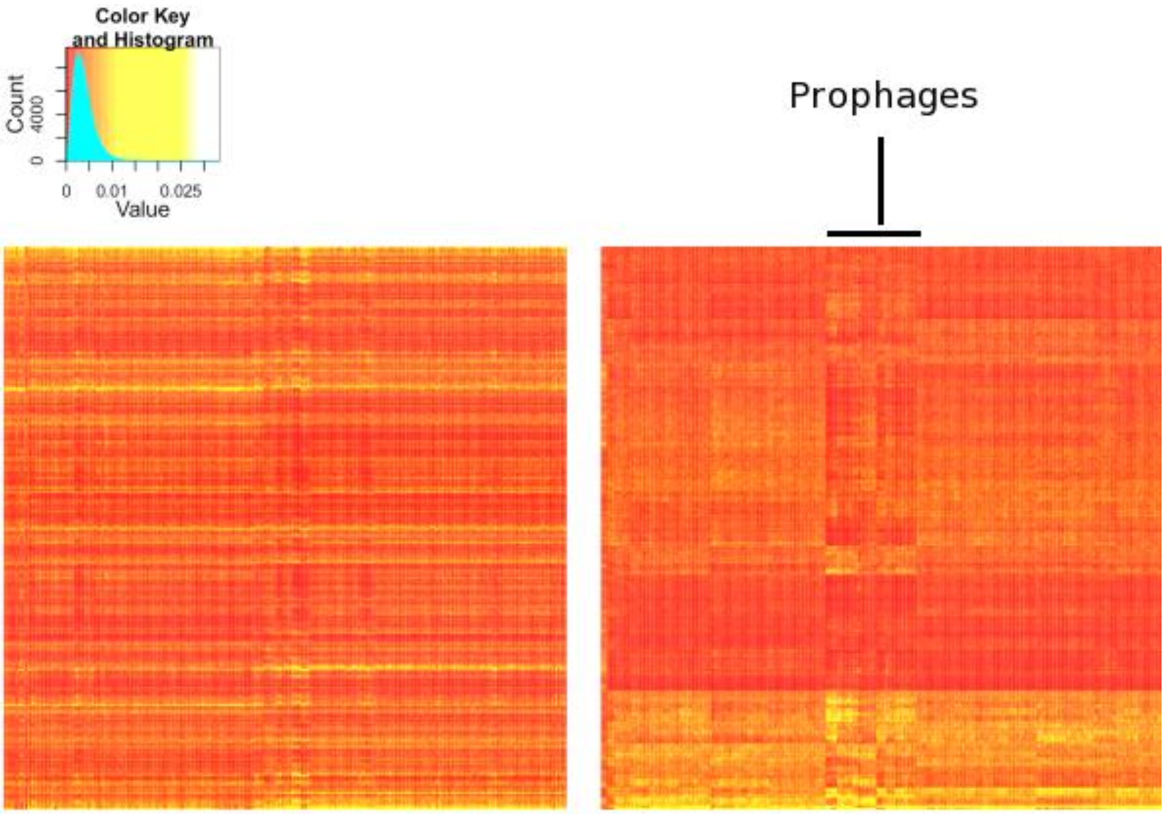

**FIGURE 1.** Oligonucleotide frequencies distributions within the *Bacillus subtilis* genome. The graphs show tetranucleotide frequencies (vertical axis) calculated from the first nucleotide to the last (horizontal axis) in the *B. subtilis* genome using a sliding window of 5 kbp from left to right. On the left graph, the position of the sliding windows is arranged in the sequential order of the genome. On the right graph, the sliding window positions are arranged according to how the tetranucleotide patterns for each sliding window are clustered. The figure clearly illustrates how the visual effect changes within the *B. subtilis* genome before (left graph) and after (right graph) clustering. It has been shown previously that the marked regions with the unusual tetranucleotide patterns in the middle on the rightmost figure are prophages. A close inspection of the leftmost graph may indicate that the prophages have been integrated at many different places in the genome; alternatively, that there have been multiple transfers even from the same host. The histogram on top shows the distribution of tetranucleotides in the *B. subtilis* genome.

drift than GC-rich genomes[20,23,59]. This tendency has support from other studies using different methods[28,35,60]. As mentioned earlier, the mutational bias in the genomes of AT-rich bacteria are assumed to be a result of low selective pressures and, possibly, lost DNA repair genes, subsequently resulting in gene loss and genome decay[27]. The pathogen *Mycobacterium leprae* is presumed to have followed such a path[61]. While AT-rich genomes are most often found in intracellular bacteria, the genomes of GC-rich bacteria are usually bigger and often found in the soil or soil-like environments[30,58]. GC-rich and soil-inhabiting bacteria have previously been found to have a more complex gene regulation system in terms of a higher number of regulators per gene than AT-rich and intracellular bacteria[29,31]. It is possible that this explanation is one of the reasons why GC-rich bacteria are more homogeneous in terms of the genomic signature than AT-rich bacteria[23]. Gene regulation in bacteria is still under extensive investigation, however, and a recent study shows that gene regulation related to metabolism in bacteria with reduced genomes appears to be more complex than initially assumed[62].

Elhai found that the maximal-order Markov chain model approximated oligonucleotide frequencies in *Escherichia coli* poorly[63]. In other words, genomic di- and trinucleotide frequencies did not approximate tetranucleotide frequencies well in the *E.coli* genome. A more complex model taking into consideration oligonucleotide usage allowing oligonucleotide patterns to be separated by several nucleotides was found to be superior. The finding that oligonucleotide frequencies of AT-rich genomes were easier to approximate than GC-rich genomes may therefore imply that nucleotide frequencies may influence AT-rich genomes over shorter ranges than in GC-rich genomes. If long-range correlations of nucleotide frequencies influence base composition more in GC-rich genomes than in AT-rich genomes, this may explain the poor approximations of the maximal-order Markov chain model in *E.coli* since it assumes short-range correlations between nucleotides.

## GENOMIC SIGNATURES: ADVANTAGES AND DRAWBACKS

The signatures from genomic DNA sequences make possible comparison of nonhomologous DNA sequences and determine the phylogenetic relationship of the host to arbitrary DNA sequences. In addition, signature variations within microbial genomes are associated with pathogenicity islands and horizontally transferred DNA since it is believed that such genes have been subjected to different evolutionary pressures[5]. The methods discussed here currently require relatively large chunks of DNA to be able to identify host organisms from arbitrary DNA sequences. An experiment was carried out where arbitrary, fixed-sized windows of genomic DNA were extracted from various genomes to examine the discriminatory power of the genomic signatures. Different sizes of the DNA chunks examined were tested, ranging from 1, 4, 8, 16, and 30 kb, and each portion of DNA was picked from randomly chosen regions in each genome. The mean AT content of each genome varied from 30 to 70%. The genomes were subsequently clustered based on dinucleotide-based genomic signatures. Not surprisingly, the groupings improved with the size of the DNA chunk used and the result of the cluster analysis based on arbitrary 30-kb DNA chunks can be observed in Fig. 2. From the same figure, it can also be seen that GC-rich genomes appear to cluster more consistently with respect to phylogeny than the AT-rich genomes. As mentioned above, genomic AT content influences the genomic signature and AT-rich genomes tend to be more affected by mutational bias than GC-rich genomes[35]. However, the size of the arbitrary DNA sequences needed to identify the host has not been examined in detail. The large size of the DNA sequences required for reliable host identification based on genomic signatures is a major drawback with the method. In addition, when applied for the detection of foreign DNA sequences, the current methods used to identify the genomic signatures can never be more specific than the size of the sliding window. In summary, although the methods discussed in the present work can be applied to assess the taxonomy, to some extent, from DNA sequences with unknown hosts, an important application in metagenomics[48], they require long sequences due to a low signal-to-noise ratio. Care should be taken, in general, when genomic signatures alone are used for taxonomic inference of microbes due to the many factors associated with the signal[24].

## PROSPECTS

The methods discussed here show that there are species-specific signals in genomic DNA sequences. The increasing number of sequenced genomes contains huge quantities of information that will require considerable computational power to analyze. Computational methods that can extract relevant information from only a fraction of a genome's DNA sequence are therefore of great importance. This ability is of great importance in metagenomics, which is becoming progressively more common and requires efficient tools to analyze the vast amounts of emerging data. The oligonucleotide frequency–based genomic signatures discussed here require relatively large amounts of genomic DNA, but it is conceivable that more advanced mathematical methods may be required, such as wavelet analysis and

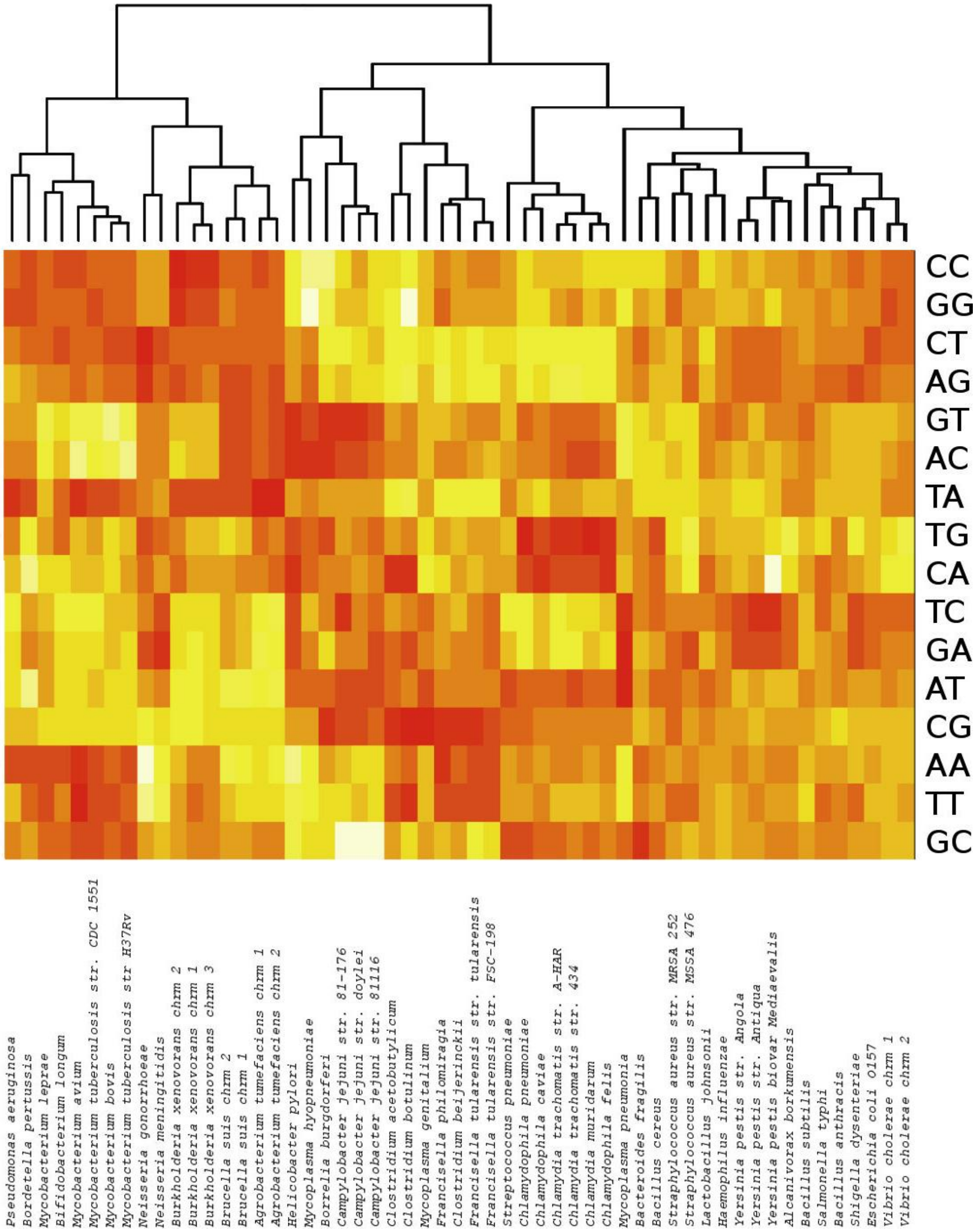

**FIGURE 2.** Cluster analysis of 50 microbes based on genomic signatures of arbitrary pieces of 30-kbp DNA. The bacteria are clustered with respect to the genomic signatures from 30 kbp of arbitrary DNA sequences taken randomly from each genome (horizontal axis). The signature of each dinucleotide can be found on the vertical axis. The degree of shading from dark to light color indicates low and high frequency of occurrence, respectively, of the dinucleotide in question compared to what is expected from genomic AT/GC content. In other words, 30 kbp of genomic DNA was randomly picked from 50 predetermined prokaryotes ranging from AT-rich mycoplasmas to the GC-rich mycobacteria. It can be seen that closely related species and strains, with the notable exception of species from genera *Mycoplasma* and *Bacillus*, tend to cluster together, while the clustering of more distantly related microbes is more arbitrary. It can also be noticed that each dinucleotide clusters together with its reverse complement, indicating similar signatures even for small (i.e., 30 kbp) contigs.

fractal-based methods[64,65,66,67,68,69,70,71], not based on oligonucleotide frequencies, but rather on individual nucleotide patterns, and that might reflect the genomic signature more effectively, giving a stronger signal and requiring shorter DNA sequences for reliable analysis. Reducing the sequence size needed to obtain a distinct genomic signature, as well as improving the signal strength, will make it possible to detect smaller horizontally transferred sequences, including pathogenicity islands in microbes. Although a weak signal can be extracted from noncoding regions, more sensitive methods might be able to extract valuable information from such regions, making methods based on genomic signatures more applicable on eukaryotic species with a low percentage of protein-coding DNA.

## ACKNOWLEDGMENTS

Funding and support provided by Prof. Gudmund Holstad and Prof. Eystein Skjerve from the Department of Food Safety and Infection Biology, Norwegian School of Veterinary Science. Thanks to Dr. Simon P. Hardy for critically drafting and revising the manuscript, and the reviewers for providing constructive comments and suggestions.
